# 2539. Towards Biomarker-Guided Model Informed Precision Dosing – A Case Study With Continuously Infused Vancomycin in Sepsis

**DOI:** 10.1093/ofid/ofad500.2156

**Published:** 2023-11-27

**Authors:** Julian Ermtraud, David Uster, Pieter De Cock, Sebastian Wicha

**Affiliations:** University of Hamburg, Hamburg, Hamburg, Germany; University of Hamburg, Hamburg, Hamburg, Germany; Ghent University Hospital, Genth, Oost-Vlaanderen, Belgium; University of Hamburg, Hamburg, Hamburg, Germany

## Abstract

**Background:**

Model-informed precision dosing can streamline the dosing individualization of vancomycin in patients with severe bacterial infections. Yet, currently utilized models are limited to predicting the pharmacokinetics (PK). In this study, a pharmacometric model was developed that described the longitudinal relationship between PK and CRP after continuous infusion of vancomycin. Subsequently, its forecasting performance was evaluated.

**Methods:**

In a clinical dataset in 67 patients with sepsis, the PK was linked to the CRP time courses using a turnover model with an effect compartment. For the forecasting analysis, scenarios with different numbers of TDM samples were tested to investigate how many are needed for valid predictions of future (unobserved) PK and CRP samples.

**Results:**

Overall, the developed pharmacometric model described the PK and CRP data well, except sudden CRP increases potentially occurring due to other effects than the treated infection. The half maximal effective concentration of vancomycin was estimated to be 20.3 mg/L (95 % CI= 18.9 - 21.5 mg/L). The usage of just one CRP concentration from routine clinical data led to imprecise predictions. The inclusion of a single vancomycin concentration improved the predictive performance, but a second plasma sample of vancomycin and CRP was required to increase accuracy and precision significantly.

**Figure:**
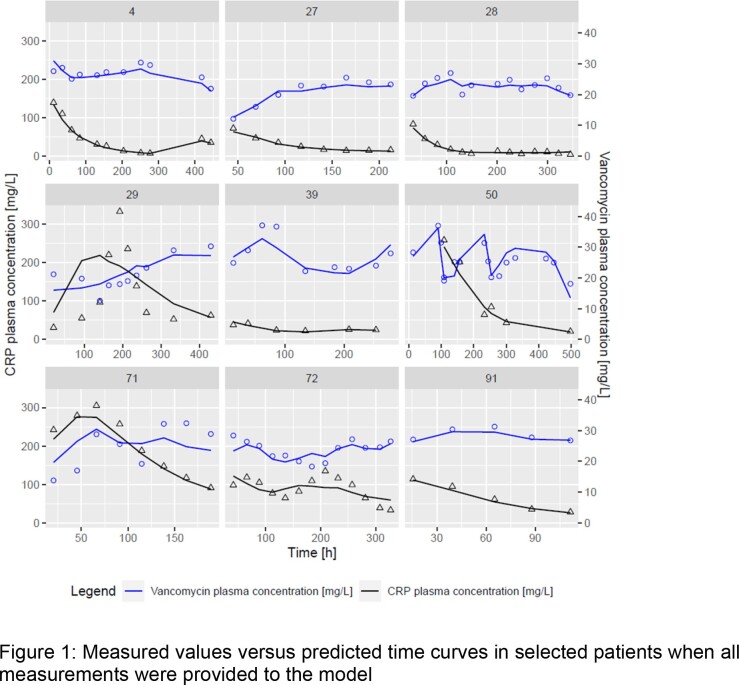

**Figure:**
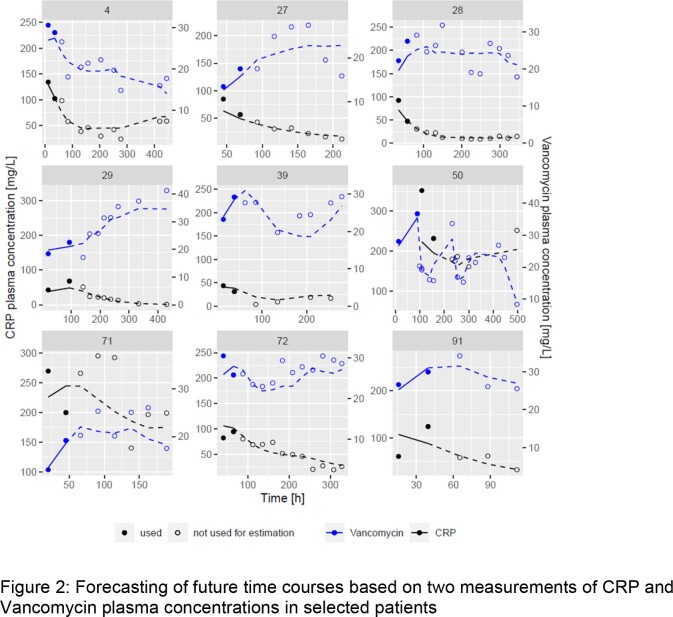

**Figure 3.**
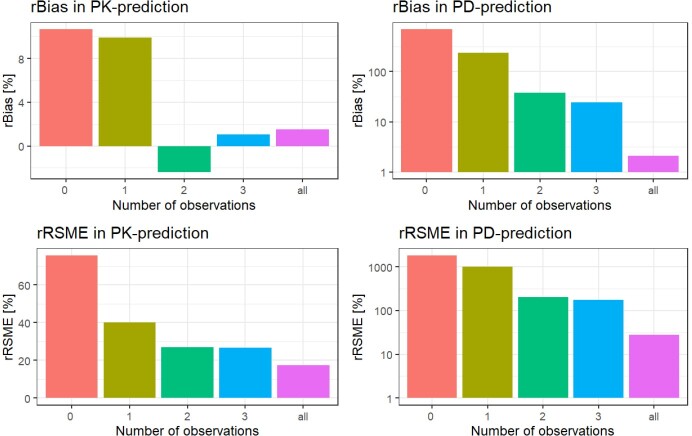
Comparison of the predictive performance of the model for the pharmacokinetics (PK) and pharmacodynamics (PD) of future (unobserved) data using the relative bias and the relative root mean squared error when different amounts of information (observed data from 0-3 or all occasions) were provided to the model.

**Conclusion:**

The present study corroborates the currently used therapeutic range for continuously infused vancomycin of 20-25 mg/L. The future tendency of the time course of up to 14 (range: 1-14) CRP measurements and vancomycin concentrations with a given vancomycin therapy regime was adequately predicted when at least two measurements of CRP and vancomycin were provided to the model, in particular if the tendency of CRP was increasing or decreasing was predicted well. These predictions are only trustworthy if there are no other events with an influence on the CRP concentration. Further biomarkers with a higher specificity for bacterial infections should be investigated or the model needs to be refined to handle such events.

**Disclosures:**

**All Authors**: No reported disclosures

